# Twenty-seven-gauge endoilluminator-assisted scleral buckling using a wide-field viewing system: Case report

**DOI:** 10.1097/MD.0000000000027206

**Published:** 2021-09-17

**Authors:** Ki Yup Nam, Hyung Bin Lim, Min Su Kim, Jung Yeul Kim

**Affiliations:** a Department of Ophthalmology, Chungnam National University Sejong Hospital, South Korea; b Department of Ophthalmology, Chungnam National University Hospital, South Korea; c Department of Ophthalmology, Chungnam National University, College of Medicine, South Korea.

**Keywords:** 27-gauge endoilluminator, case report, scleral buckle, wide-field viewing system

## Abstract

**Rationale:**

We report a new scleral buckling technique using a 27-gauge endoilluminator and a wide-field viewing system to overcome the limitations of conventional indirect ophthalmoscope-methods and “chandelier-assisted” surgery.

**Patient concerns:**

A 26-year-old female patient visited the local clinic for floaters and lower visual field defects in her left eye that had occurred 5 days prior.

**Diagnoses:**

On fundus examination, upper retinal detachment without macular involvement and an atrophic hole was observed in her left eye.

**Interventions:**

Under general anesthesia and after perilimbal conjunctival incision, extraocular muscle isolation, and traction with black silk, a 27-gauge trocar-cannula was inserted 90° away from the retinal break, 4 mm away from the limbus. Under wide-field viewing using a contact lens, the fundus was observed through a surgical microscope. Retinal break was evaluated and cryopexy was performed with careful movement of the endoilluminator, paying attention to damage to the lens. The surgeon could accurately and freely control the direction of the illumination tip to obtain a brighter view of the region of interest.

**Outcomes:**

There were no complications associated with trocar cannula incision or the illuminator. The retina was successfully reattached.

**Lessons:**

Twenty seven gauge endoilluminator-assisted scleral buckling is an easy and safe procedure and provides better control over and free adjustment of the light direction, thus overcoming the limitations of chandelier-assisted surgery.

## 1 Introduction

Scleral buckling is a basic surgical treatment method for rhegmatogenous retinal detachment. During this surgery, observing the retina is essential. Traditionally, an indirect ophthalmoscope is used to observe the fundus; however, the images of an indirect ophthalmoscope are reversed, making it difficult to perform surgical procedures. In addition, the repeated application and removal of the instrument during surgery is inconvenient and time-consuming.

**Figure 1:**
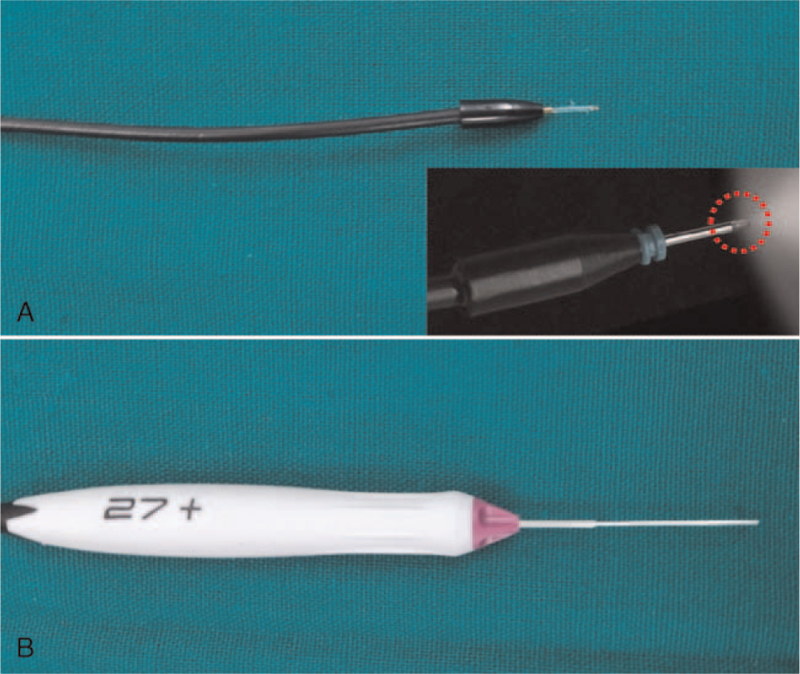
The structure of 25 gauge chandelier (A) and 27 gauge endo-illuminator (B). After insertion into the cannula, the exposed optical tip of the chandelier is very short (red dotted circle).

Recently, scleral buckling has been conducted using intraocular illumination and wide-field viewing systems. Twenty five and 27-gauge chandelier illuminations have been used in previous studies.^[1–8]^ These methods overcome the inconvenience of using an indirect ophthalmoscope. However, the optic tip of a chandelier illuminator is very short; thus, it can be difficult to focus the illumination for viewing the region of interest (Fig. 1).

Therefore, we wanted to identify a method to focus the light on the desired area by controlling the direction and distance of the illumination more freely. Here, we introduce a new technique for scleral buckling using a 27-gauge endoilluminator and a wide-field viewing system to overcome the limitations of conventional indirect ophthalmoscope-methods and “chandelier-assisted” surgery.

## 2 Case presentation

The protocol for this research was approved by the Institutional Review Board of Chungnam National University Hospital, and the study adhered to the tenets of the Declaration of Helsinki. Consent to publish this case was obtained from the patient.

A 26-year-old female patient visited a local clinic for floaters and lower visual field defects in her left eye that had occurred 5 days prior. She was referred to our ophthalmology clinic due to retinal detachment. On fundus examination, upper retinal detachment without macular involvement with an atrophic hole was observed in her left eye. The best-corrected visual acuity was 1.0 in both eyes. There were no other anterior segment abnormalities. On fundus examination, an atrophic hole was found at the 2 o’clock position on the peripheral retina, and lattice degeneration lesions were observed near the hole and at 7 o’clock.

**Figure 2:**
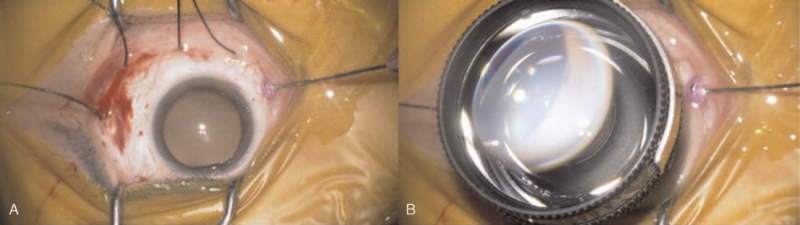
Insertion of the 27-gauge endo-illuminator and wide-field contact lens placement on the cornea. The patient had a break in the inferior retinal periphery (5 o’clock position). (A) A 27-gauge trocar-cannula was inserted 4 mm from the scleral limbus, about 90° away from the retinal break. (B) Then a wide-field contact lens was placed on the cornea.

**Figure 3:**
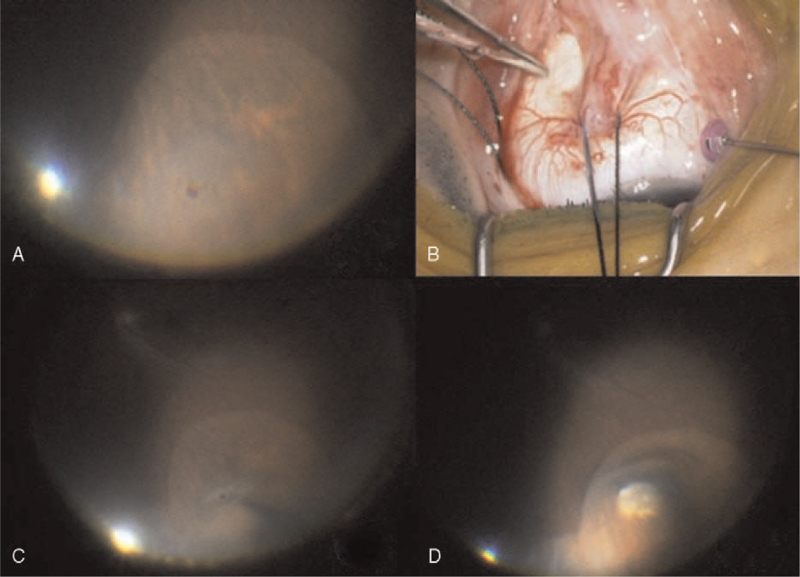
Identification of the exact location of the retinal break and cryopexy. (A–C) A scleral point corresponding to the site of the retinal break is held with tooth forceps and inwardly depressed. (D) After confirmation of the scleral point, cryopexy was performed. The endo-illuminator illuminated the region of interest well.

**Figure 4:**
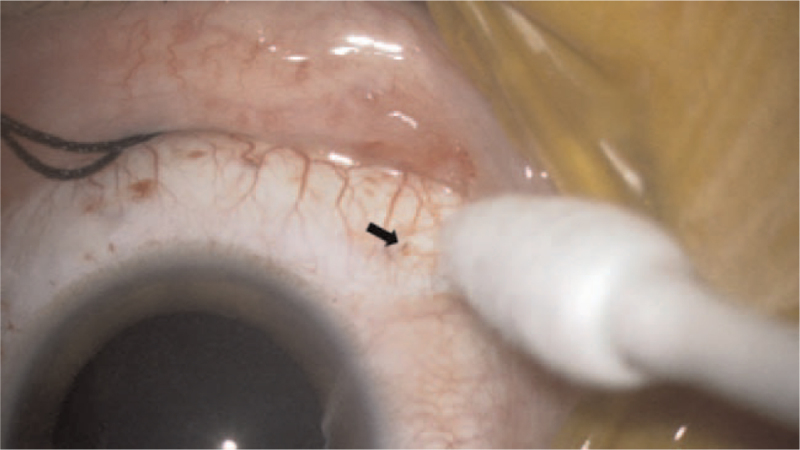
Removal of the 27-gauge cannula. After removing the cannula, the incision site was compressed with blunt forceps. No vitreous leakage was observed. The black arrow shows the site of the 27-gauge cannula removal.

Under general anesthesia, the procedures were performed using a surgical microscope. With a perilimbal conjunctival incision, the sclera was exposed, the extraocular muscles were hooked at the insertion, and traction sutures (4/0 black silk) were placed. An oblique incision with a 27-gauge-valved trocar cannula (Alcon, Chandelier lighting system, Fort Worth, TX, USA) was made 90° away from the retinal break, 4 mm posterior from the limbus. Then, a 27-gauge endoilluminator was inserted through the cannula. A wide-field contact lens (Mini Quad; Volk, Mentor, OH) was placed on the cornea, and the fundus was observed using a surgical microscope (Fig. 2). The image inverter allowed for an upright image of the fundus. The retinal break location was identified, and the surgeon marked the spot on the sclera (Fig. 3). Cryopexy was performed at the marked site. During these procedures, the surgeon could easily control the direction of the illumination tip to view the region of interest under brighter conditions. The surgeon was also careful not to touch the lens. To move the eyeball and position the silicone sponge, the endoilluminator was removed. As it was a valved cannula type, there was no need for further management of leakage from the cannula. A silicone sponge was secured to the sclera with a mattress suture using 5 to 0 Ethibond. After adjusting the intraocular pressure by performing an anterior chamber paracentesis, the endoilluminator was reinserted. The surgeon checked the position and height of the buckle by observing the fundus with the wide-field contact lens, and, while controlling the direction of illumination, the peripheral retina other than retinal detachment area was checked by scleral depression. The endoilluminator and cannula were removed sequentially, and the site was compressed using blunt forceps to close the scleral incision (Fig. 4). Surgery was completed by closing the conjunctiva.

On postoperative day 1, the hole was closed on the elevated buckle according to fundus examination. One month after surgery, the retina remained flat.

## 3 Discussion

Although an indirect ophthalmoscope is conventionally used for scleral buckling, the fundus images are reversed. Repetitive mounting and removal is inconvenient, time-consuming, and poses a risk of infection. Recently, to eliminate the disadvantages of using an indirect ophthalmoscope during scleral buckling procedures, various methods using intraocular illumination and wide-field viewing systems under a surgical microscope have been reported. With these methods, surgeons can reduce the inconvenience of using an indirect ophthalmoscope, such as to observe the fundus upright and have control over the image size.

Aras et al^[1]^ first reported scleral buckling using a 25-gauge torpedo-style chandelier light (Alcon) and a non-contact wide-field viewing system (BIOM; Oculus, Germany). The torpedo light was inserted via uncannulated sclerotomy.

Subsequently, several case series of scleral buckling procedures using a small-gauge chandelier with a transscleral cannula and non-contact or contact-type wide-viewing system have been reported.^[2]^ Nam et al^[3]^ and Nagpal et al^[4]^ used a 25-gauge chandelier (Alcon) contact lens (Mini Quad) and a wide-field viewing system. Kita et al^[5]^ used a Resight system (Carl Zeiss Meditec AG, Jena, Germany). Yokoyama et al^[6]^ and Caprossi et al^[7]^ applied an uncannulated 27-gauge chandelier (Eckard TwinLight Chandelier; DORC International, Zuidland, The Netherlands) and cannula-based 27-G chandelier (Alcon), respectively. Good surgical results have been obtained with these techniques, and no specific complications associated with the chandelier have been cited. In a large case series, Inami et al^[8]^ performed scleral buckling using a 25-gauge cannulated chandelier in 79 patients, and achieved a primary success rate of 92.4%; there were 2 cases of intraoperative complications associated with the chandelier. Specifically, an iatrogenic retinal break developed during removal of the chandelier in 1 case, and lens touch occurred during cryopexy in the other case; however, the cataract did not progress afterwards.

Overall, according to previous reports, scleral buckling procedures using a small-gauge chandelier and a wide-field viewing system seems to be an easy, safe and efficient method. The good visualization allows surgeons to check the peripheral retina more precisely. In several studies, the authors reported that they could find the retinal breaks intraoperatively, which were not identified in preoperative examinations.^[5,6]^

Due to these advantages, our group has performed 25-gauge chandelier assisted scleral buckling procedures. Over time, however, we realized the limitations of using the chandelier. The optic tip is too short, and surgeons cannot move the position of illumination freely; therefore, it is difficult to focus the illumination on the desired area. Also, there is a risk of lens touch when tilting the eye, as described in the abovementioned report.

In an effort to address these limitations, we attempted to use a 27-gauge valved cannula-based endoilluminator. The most important advantage of using an endoilluminator is that surgeons can move the illuminator at will, thus allowing evaluation of the retina area under brighter illumination. Lens touch is a concern due to the long shaft of the endoilluminator; however, in our experience, there is less risk of damage compared to that when using a chandelier, as the endoilluminator can be moved carefully, similar to what is required when performing a lens-sparing vitrectomy. Additionally, the 27-gauge endoilluminator is sufficiently small to close the scleral incision by simply pressing it with a cotton swab. However, sudden tilting of the eyeball can be problematic; thus, surgeons should pay close attention to this as the endoilluminator shaft may slide in an unexpected way. To evaluate other possible complications, more experience with this method is needed.

In conclusion, scleral buckling using a 27-gauge endoilluminator and a wide-field viewing system shows great promise for overcoming the limitations of chandelier-assisted surgery.

## Author contributions

**Conceptualization:** Jung Yeul Kim.

**Data curation:** Ki Yup Nam, Hyung Bin Lim, Min Su Kim.

**Investigation:** Ki Yup Nam, Jung yeul Kim.

**Methodology:** Ki Yup Nam.

**Supervision:** Jung yeul Kim.

**Writing – original draft:** Ki Yup Nam, Jung Yeul Kim.

**Writing – review & editing:** Ki Yup Nam, Hyung Bin Lim, Min Su Kim, Jung Yeul Kim.
